# A predictive coding approach to modelling the perceived complexity of popular music drum patterns

**DOI:** 10.1016/j.heliyon.2023.e15199

**Published:** 2023-04-07

**Authors:** Olivier Senn

**Affiliations:** Music Department, Lucerne University of Applied Sciences and Arts, Arsenalstrasse 28a, 6010, Kriens, Switzerland

**Keywords:** Predictive coding, Music perception, Complexity, Popular music, Drums, Modelling, Rhythm

## Abstract

This study presents a method to estimate the complexity of popular music drum patterns based on a core idea from predictive coding. Specifically, it postulates that the complexity of a drum pattern depends on the quantity of surprisal it causes in the listener. Surprisal, according to predictive coding theory, is a numerical measure that takes large values when the perceiver's internal model of the surrounding world fails to predict the actual stream of sensory data (i.e. when the perception surprises the perceiver), and low values if model predictions and sensory data agree. The proposed new method first approximates a listener's internal model of a popular music drum pattern (using ideas on enculturation and a Bayesian learning process). It then quantifies the listener's surprisal evaluating the discrepancies between the predictions of the internal model and the actual drum pattern. It finally estimates drum pattern complexity from surprisal. The method was optimised and tested using a set of forty popular music drum patterns, for which empirical perceived complexity measurements are available. The new method provided complexity estimates that had a good fit with the empirical measurements ($R^{2}=.852$). The method was implemented as an *R* script that can be used to estimate the complexity of popular music drum patterns in the future. Simulations indicate that we can expect the method to predict perceived complexity with a good fit ($R^{2}\geq .709$) in 99% of drum pattern sets randomly drawn from the Western popular music repertoire. These results suggest that surprisal indeed captures essential aspects of complexity, and that it may serve as a basis for a general theory of perceived complexity.

## Highlights

• Surprisal-based model of perceived complexity in popular music drum patterns.
• Computational method to estimate drum pattern complexity.
• Good model fit indicates that predictive coding is a possible basis for a theory of complexity.

## 1 Introduction

The *complexity* of art or music has long been understood as an essential aesthetic dimension ([[1], [2], [3], [4]]), and there is an abundant body of research that investigates the relationship between the complexity of artifacts and how much observers appreciate them (for an overview, see Refs. [5,6]). Researchers seem to agree that the complexity of an artifact can be expressed as a quantity (and thus measured), and that it is sensible to state that one artifact is more complex than another. The concept of complexity in art is difficult to grasp, in spite of its frequent use. It seems to have an objective and a subjective component: on one hand, complexity is rooted in the artifact itself, in its objective properties, in the relationships between its parts and, as Pressing described it, in the “existence of structure on many scales or levels” ([7], p. 1). On the other hand, complexity is also experienced subjectively by the person perceiving the artifact: understanding the structure of the artifact can be seen as a problem that needs to be solved, and the complexity of the artifact has been “defined as the minimal cost of computing an approximate solution to the problem” ([7], p. 2).

In the domain of music, most measures of musical complexity emphasise objective aspects and concentrate predominantly on rhythmic complexity. Some objective measures were based on rhythm-related music theoretic concepts such as syncopation ([8,9]), rhythmic oddity ([10]), metric complexity ([11]), offbeatness or note-to-beat distance ([12]). Other approaches involve information theoretic and probabilistic concepts, e.g. Shannon entropy, excess entropy, entropy rate, cross-entropy, or Kolmogorov complexity ([[13], [14], [15]]).

This study proposes a new approach to measuring complexity, based on ideas from predictive coding (PC), that combines objective and subjective aspects. PC is a theory that aims at explaining cognitive functions such as perception, learning, and action ([16,17]); it may potentially provide a unifying framework for the cognitive sciences ([18]). PC is based on the idea that an organism's brain is essentially a prediction machine which compares incoming sensory data with the predictions of an internal model. The organism aims at avoiding discrepancies between the sensory data and the predictions, because these discrepancies may indicate an unexpected danger from the outside world. The organism has two possibilities to harmonise the sensory data with the predictions: it can either update the internal model in order to better predict the sensory data in the future (*learning*) or it can influence the outside world and thus supress discrepant sensory data (*action*, see Ref. [16], p. 2). During the last two decades, PC theory was formulated in rigorous mathematical terms ([19,20]), and it was successfully applied to model cognitive functions in a variety of contexts and disciplines (for a comprehensive overview, see the preprint by Millidge et al. [21]).

This study aims at modelling the complexity of drum patterns from Western popular music using the PC concept of *surprisal*. Surprisal is a non-negative metric that, at each moment in time, quantifies the surprise triggered by the discrepancy between sensory data and the predictions of the internal model. This study hypothesises that the subjectively perceived complexity of a drum pattern depends on the total quantity of surprisal it causes in the listener. The quantity of surprisal will be calculated depending on the objective properties of the pattern (which represents the *sensory data*), on the expectations of the listener determined by their experience and cultural background (the listener's *internal model*), and on a mechanism that detects the regularities of the pattern as it unfolds and adapts the internal model to reduce surprisal in the future (*learning*).

The modelling work in this study relies on a set of forty drum patterns from the Western popular music repertoire that was recently presented in a preprint by Senn et al. ([22]) and that was drawn from a larger corpus of drum patterns (*Lucerne Groove Research Library*, www.grooveresearch.ch). The forty drum patterns are paired with reliable perceived complexity measures, established empirically in a listening experiment. The success of the modelling effort, and thus the merit of the PC-related ideas to define complexity, will be judged based on the agreement between estimated complexity and the empirical measures of perceived complexity. This agreement is measured by the $R^{2}$ effect size (the square of the Pearson product moment correlation). The model will be successful if it outperforms the benchmark of $R^{2}=.491$ set by the *Index of Syncopation* developed by Witek et al. ([9]), an objective measure that is frequently used to estimate drum pattern complexity, and the *Revised Index of Syncopation* by Hoesl and Senn ([23]) derived from Witek et al.’s, which achieves $R^{2}=.560$ (for the fit of these existing measures with the empirical measurements, see Ref. [22], p. 18).

This study is not the first to use the PC framework in order to explain phenomena relevant to music cognition and rhythm. A series of neuroscientific studies ([[24], [25], [26], [27]]) used electroencephalography to investigate how single surprising rhythmic events are processed in the brain. Vuust et al. [24] showed that a surprising event triggered the mismatch negativity reaction (MMNm, ca. 110–130 ms after stimulus onset) and a P3am response (ca. 80 ms after the MMNm). They interpreted the MMNm reaction as the brain's detection of a discrepancy (which can be understood as a neural equivalent of *surprisal*), and the P3am as an evaluation of this discrepancy. Vuust et al. developed their ideas into a coherent theory called *Predictive Coding of Rhythmic Incongruity* or *PCRI* ([25,26]) to which Lumaca et al. ([27]) added further evidence and nuance. The current study shares some fundamental ideas on PC with these previous studies, but uses the ideas for different purposes. The neuroscientific studies focused on the implementation of PC-related physiological processes in the central nervous system, based on single surprising events. In the current study, the emphasis lies on the development of a mathematically sound measure of pattern complexity based on surprisal without considering the neural substrate. The neuroscientific studies operate on the lower implemental and algorithmic levels of Marr's hierarchy (see Refs. [28,29], p. 185) whereas the current effort can be located on the hierarchy's upper computational level.

## 2 Materials and methods: Perceived complexity of forty popular music drum pattern stimuli

All popular music drum patterns used in this study were taken from a corpus called the *Lucerne Groove Research Library* (www.grooveresearch.ch). The library currently consists of 251 popular music drum patterns in 4/4 common time that have a duration of 8 bars. Each pattern was originally played by a renowned drummer on a full-band recording in one of the major Western popular music styles (rock, pop, funk, soul, heavy metal, rock'n'roll, disco and others). The drum patterns were carefully reconstructed by Lorenz Kilchenmann as audio stimuli using drum samples, based on transcriptions and exact timing measurements by Toni Bechtold and Florian Hoesl. The majority of these stimuli were first used in a 2018 groove study by Senn et al. [30], which also provides more information about the corpus and stimuli preparation (see also published data and meta-data on the corpus in Ref. [31]).

In their preprint, Senn et al. ([22]) used forty of the library's drum patterns (see Table 1) to create a stimuli set with perceived complexity measures. They selected patterns that cover a wide complexity range (as measured by the *Index of Syncopation*, see Ref. [9]) and that have a parsimonious instrumentation with bass drum, snare drum, and cymbals. Senn et al. ([22]) shortened the patterns to 4 bars (plus the first beat of bar five). These forty patterns and the complexity estimates will be used in this study to train and test the model. The remaining 211 patterns of the library will be used to inform the model about the expectations of an enculturated listener with respect to the structure of popular music drum patterns.

**Table 1:** *Perceived complexity* (Bradley-Terry coefficients ${\hat{\beta }}_{i}$, see Ref. [22], p. 13 ) and *estimated complexity* (${\hat{\gamma }}_{i}$) for each of the 40 drum pattern stimuli.

Stimulus (i)	Title	Perceived Complexity (${\hat{\beta }}_{i}$)	Estimated Complexity (${\hat{\gamma }}_{i}$)
1	A Kind Of Magic	0.400	0.728
2	(Sittin’ On) The Dock Of The Bay	0.408	0.653
3	Smells Like Teen Spirit	0.476	0.444
4	Boogie Wonderland	0.573	0.947
5	Vultures	0.784	0.611
6	Kashmir	1.182	1.048
7	Street Of Dreams	1.210	0.969
8	Change The World	1.230	1.511
9	Let's Dance	1.263	1.582
10	Space Cowboy	1.415	1.424
11	I Feel For You	1.632	1.851
12	Virtual Insanity	1.859	1.745
13	Bravado	1.902	2.391
14	Let's Go Dancing	1.951	1.991
15	Discipline	2.091	1.744
16	Pass The Peas	2.151	1.578
17	The Pump	2.189	2.809
18	Roxanne	2.216	1.740
19	Dreamin’	2.413	1.840
20	Soon I'll Be Loving You Again	2.511	2.284
21	Summer Madness	2.530	2.678
22	Listen Up!	2.586	3.148
23	Jungle Man	2.752	3.272
24	Shake Everything You Got	3.051	3.124
25	Chicken	3.052	3.588
26	Cissy Strut	3.080	3.376
27	Far Cry	3.120	3.247
28	Alone + Easy Target	3.130	2.356
29	Soul Man	3.263	3.681
30	Ain't Nobody	3.300	3.371
31	Diggin' On James Brown	3.314	2.975
32	In The Stone	3.342	3.368
33	Southwick	3.360	3.691
34	You Can Make It If You Try	3.447	3.230
35	The Dump	3.464	3.984
36	Killing In The Name Of	3.564	2.197
37	Cold Sweat	3.763	2.824
38	Hyperpower	3.902	4.027
39	Rock Steady	4.394	4.620
40	Jelly Belly	4.701	4.327

Senn et al. ([22]) estimated the complexity of the forty drum patterns on the basis of a listening experiment with an incomplete pairwise comparison design, in which 220 participants judged the relative complexity of the stimuli in a total of 4400 pairwise comparison trials. The data from the experiment were analysed using the Bradley-Terry probability model. This resulted in the *perceived complexity* (${\hat{\beta }}_{i}$) estimates listed in Table 1.

The Bradley-Terry estimates ${\hat{\beta }}_{i}$ have a clear probabilistic interpretation: they allow to calculate the probability that one drum pattern is considered to be more complex than another drum pattern when judged in a pairwise comparison trial by a random member of the listener population. The estimated success probability ${\hat{\Pi }}_{ij}$ that stimulus i is perceived as being more complex than stimulus j is:

(1) $${\hat{\Pi }}_{ij}=\frac{e^{{\hat{\beta }}_{i}-{\hat{\beta }}_{j}}}{1+e^{{\hat{\beta }}_{i}-{\hat{\beta }}_{j}}}$$

where the *perceived complexity* measures ${\hat{\beta }}_{i}$ and ${\hat{\beta }}_{j}$ are equal to the Bradley-Terry coefficients corresponding to stimuli i and j ([32], p. 265). To make an example: the probability ${\hat{\Pi }}_{3,23}$ that stimulus 3 (“Smells Like Teen Spirit”) is heard as being more complex than stimulus 23 (“Jungle Man”) is estimated at:

$${\hat{\Pi }}_{3,23}=\frac{e^{{\hat{\beta }}_{3}-{\hat{\beta }}_{23}}}{1+e^{{\hat{\beta }}_{3}-{\hat{\beta }}_{23}}}=\frac{e^{0.476-2.752}}{1+e^{0.476-2.752}}≅0.093\text{.}$$

Here, we used the ${\hat{\beta }}_{3}=0.476$ and ${\hat{\beta }}_{23}=2.752$ coefficients from Table 1. We can expect the “Smells Like Teen Spirit” drum pattern (stimulus 3) to be considered more complex than the “Jungle Man” drum pattern (stimulus 23) in only 9.3% of pairwise comparison trials. Conversely, the probability that “Jungle Man” is considered more complex than “Smells Like Teen Spirit” is:

$${\hat{\Pi }}_{23,3}=\frac{e^{{\hat{\beta }}_{23}-{\hat{\beta }}_{3}}}{1+e^{{\hat{\beta }}_{23}-{\hat{\beta }}_{3}}}=\frac{e^{2.752-0.476}}{1+e^{2.752-0.476}}≅0.907\text{.}$$

“Jungle Man” can be expected to win 90.7% of the trials against “Smells Like Teen Spirit”. Since no ties are allowed in the pairwise contests, it is generally true that ${\hat{\Pi }}_{ij}+{\hat{\Pi }}_{ji}=1$. Solving equation (1) for ${\hat{\beta }}_{i}-{\hat{\beta }}_{j}$ yields:

(2) $${\hat{\beta }}_{i}-{\hat{\beta }}_{j}=\log\left(\frac{{\hat{\Pi }}_{ij}}{{\hat{\Pi }}_{ji}}\right)=logit\left({\hat{\Pi }}_{ij}\right)\text{.}$$

The difference ${\hat{\beta }}_{i}-{\hat{\beta }}_{j}$ is the log-odds or logit of the probability ${\hat{\Pi }}_{ij}$ that stimulus i is judged to be more complex than stimulus j by a random member of the listener population.

## 3 Defining estimated complexity based on surprisal

### 3.1 Surprisal and effort

During its lifetime, a sentient organism is subject to a stream of sensory information s(t) that varies over time t. According to predictive coding theory, the organism has an internal probabilistic generative model m(t), which expects s(t) with a certain probability. The intensity of the organism's surprise, or *surprisal*, at any point in time can be defined as follows (see also the definitions of surprisal in Refs. [16], p. 2, and [33], p. 64):

(3) Surprisal(t)=f(t)=−log[p(s(t)|m(t))].

*Surprisal* is the negative natural logarithm of the probability to experience the sensation s(t) at time t, given that the internal model is currently in state m(t). This definition of surprisal uses the prediction error between what the internal model expects to happen at time t and what actually does happen at time t in order to quantify the strength of the listener's surprise. This is one of several possible ways to mathematically express the everyday experience of being surprised. It falls into the category of “probabilistic mismatch surprise measures” in the taxonomy of Modirshanechi et al. ([34]).

Since probabilities are in the range [0,1], *surprisal* has range [0,∞). When a sensation happens that was predicted by the internal model with high probability, *surprisal* takes a positive value near zero. Conversely, if a sensation happens that had a low probability according to the internal model m(t), surprisal takes a high positive number.

**Fig. 1:**
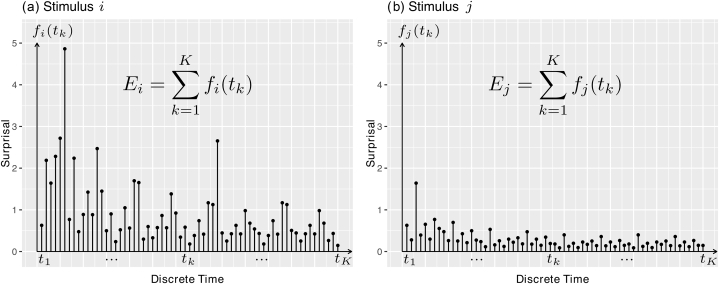
Surprisal functions of stimuli i and j, and the calculation of the associated efforts $E_{i}$ and $E_{j}$.

Let us assume, stimulus i triggers the surprisal function $f_{i}$ in a listener, as visualised in Fig. 1 (a). We define the *effort* $E_{i}$ it takes a listener to cognitively process stimulus i as the sum of the surprisal values between the beginning of the stimulus at time $t_{1}$ until it ends at $t_{K}$:

(4) $$E_{i}=\sum_{k=1}^{K}f_{i}\left(t_{k}\right)\text{.}$$

Note that, here and throughout this entire study, the time domain is understood as consisting of K discrete, equidistant time points $t_{1},t_{2},\ldots ,t_{K}$ at which the values of the surprisal function will be evaluated. For stimulus i in Fig. 1 (a), the surprisal values are relatively large, whereas for stimulus j, Fig. 1 (b) shows lower surprisal values. Consequently, stimulus i is associated with a greater effort than j. Overall, surprising stimuli will be associated with a great effort.

### 3.2 Estimated complexity

In this paragraph, we develop the *estimated complexity* values ${\hat{\gamma }}_{i}$ in several steps, using concepts such as the effort $E_{i}$ and the success probability ${\hat{\Pi }}_{ij}$ that have been introduced earlier. The goal of this paragraph is to define *estimated complexity* ${\hat{\gamma }}_{i}$ in such a way that it is equivalent to *perceived complexity* ${\hat{\beta }}_{i}$ (empirically measured in Ref. [22]), and that, consequently, ${\hat{\gamma }}_{i}$ and ${\hat{\beta }}_{i}$ have the same probabilistic interpretation.

First, we define an expression for the success probability ${\hat{\Pi }}_{ij}$ that stimulus i wins a pairwise comparison trial against stimulus j (and, conversely, the probability ${\hat{\Pi }}_{ji}$ that stimulus j wins against i) as follows:

(5) $${\hat{\Pi }}_{ij}=\frac{E_{i}^{C}}{E_{i}^{C}+E_{j}^{C}}\;\;\;and\;\;\;{\hat{\Pi }}_{ji}=\frac{E_{j}^{C}}{E_{i}^{C}+E_{j}^{C}}\text{,}$$

where C>0 is a constant that needs to be estimated, and $E_{i}$, $E_{j}$ are the efforts associated with the two stimuli. Note that ${\hat{\Pi }}_{ij}$ and ${\hat{\Pi }}_{ji}$ are necessarily in the range [0,1] adequate for probabilities, because $E_{i},E_{j},C>0$. Also:

$${\hat{\Pi }}_{ij}+{\hat{\Pi }}_{ji}=\frac{E_{i}^{C}}{E_{i}^{C}+E_{j}^{C}}+\frac{E_{j}^{C}}{E_{i}^{C}+E_{j}^{C}}=\frac{E_{i}^{C}+E_{j}^{C}}{E_{i}^{C}+E_{j}^{C}}=1\text{.}$$

Since there are no ties in the trials, the two probabilities must add up to 1. Also, if $E_{i}=E_{j}$, then:

$${\hat{\Pi }}_{ij}=\frac{E_{i}^{C}}{E_{i}^{C}+E_{j}^{C}}=\frac{E_{j}^{C}}{E_{i}^{C}+E_{j}^{C}}={\hat{\Pi }}_{ji}=0.5\text{.}$$

If the efforts associated with stimuli i and j are equal, then the success probabilities are equal at ${\hat{\Pi }}_{ij}={\hat{\Pi }}_{ji}=0.5$ as well. We define the *estimated complexities* ${\hat{\gamma }}_{i}$ and ${\hat{\gamma }}_{j}$ of stimuli i and j to be:

(6) $${Estimated\;Complexity}_{i}={\hat{\gamma }}_{i}=\log\left(E_{i}^{C}\right)+D=C\;\log\left(E_{i}\right)+D$$

(7) $${Estimated\;Complexity}_{j}={\hat{\gamma }}_{j}=\log\left(E_{j}^{C}\right)+D=C\;\log\left(E_{j}\right)+D$$

where D∈R is another constant. The difference ${\hat{\gamma }}_{i}-{\hat{\gamma }}_{j}$ can then be expressed as follows, using (6), (7), (5):

(8) $${\hat{\gamma }}_{i}-{\hat{\gamma }}_{j}=\left(C\;\log\left(E_{i}\right)+D\right)-\left(C\;\log\left(E_{j}\right)+D\right)=\log\left(E_{i}^{C}\right)-\log\left(E_{j}^{C}\right)=\log\left(\frac{E_{i}^{C}}{E_{j}^{C}}\right)=\log\left(\frac{\frac{E_{i}^{C}}{E_{i}^{C}+E_{j}^{C}}}{\frac{E_{j}^{C}}{E_{i}^{C}+E_{j}^{C}}}\right)=\log\left(\frac{{\hat{\Pi }}_{ij}}{{\hat{\Pi }}_{ji}}\right)=logit\left({\hat{\Pi }}_{ij}\right)\text{.}$$

From equation (8) follows that the ${\hat{\gamma }}_{i}$ have the same interpretation as the Bradley-Terry coefficients ${\hat{\beta }}_{i}$ shown in equation (2):

(9) $${\hat{\gamma }}_{i}-{\hat{\gamma }}_{j}=logit\left({\hat{\Pi }}_{ij}\right)={\hat{\beta }}_{i}-{\hat{\beta }}_{j}\text{.}$$

This implies that *perceived complexity* ${\hat{\beta }}_{i}$ and *estimated complexity* ${\hat{\gamma }}_{i}$ are equivalent. Consequently, we can calculate the probability ${\hat{\Pi }}_{ij}$ in analogy to equation (1):

$${\hat{\Pi }}_{ij}=\frac{E_{i}^{C}}{E_{i}^{C}+E_{j}^{C}}=\frac{e^{{\hat{\gamma }}_{i}-{\hat{\gamma }}_{j}}}{1+e^{{\hat{\gamma }}_{i}-{\hat{\gamma }}_{j}}}\text{.}$$

The constant C scales the efforts $E_{i}$ in order to allow for this probabilistic interpretation. Strictly speaking, the constant D is not necessary, since it is canceled in the difference ${\hat{\gamma }}_{i}-{\hat{\gamma }}_{j}$. However, it allows us to transpose the ${\hat{\gamma }}_{i}$ in such a way that they take values in the same range as the Bradley-Terry ${\hat{\beta }}_{i}$ and are thus easier to interpret.

With the definition of *estimated complexity* in equations (6), (7), two goals have been achieved: firstly, the *estimated complexity* ${\hat{\gamma }}_{i}$ of stimulus i is entirely based on the effort $E_{i}$ to perceive stimulus i (and thus on the surprisal function associated with stimulus i) plus on two constants. Secondly, equation (9) shows that the *estimated complexity* ${\hat{\gamma }}_{i}$ has the same interpretation as the *perceived complexity* of stimulus i expressed by the Bradley-Terry coefficients ${\hat{\beta }}_{i}$.

## 4 Theory and results

### 4.1 Modelling drum pattern complexity

We now move on to modelling complexity. In order to simplify, we make a few assumptions: Firstly, we consider the time dimension to be a grid of discrete 16^th^ note positions. In any 4-bar pattern, there are 16 sixteenth notes for each of the 4 bars, and the pattern ends on the first beat of bar 5. This results in K=65 metric positions on which events may happen. Secondly, we understand each drum pattern to consist of L=3 instrumental layers: bass drum (l=1), snare drum (l=2), and cymbals (l=3). The forty drum patterns that are used for modelling in this study satisfy this second assumption, since they only feature bass drum, snare drum and different cymbals. Toms and other percussion instruments were explicitly excluded in the selection of these patterns (for pattern selection, see Ref. [22]). Thirdly, information on event loudness is discarded.

The discrete time and instrumentation dimensions allow to encode all relevant information in matrix form. The modelling process can then be carried out using methods of linear algebra. It consists of the following five steps:

1. *Encoding the drum pattern matrix*$S_{i}$: In this step, the drum pattern *i* is encoded as a matrix in binary form. An onset is encoded 1 and the absence of an onset is encoded 0 for each time point k and each instrumental layer l. This information represents the sensory data, and it is collected in the 65×3 matrix $S_{i}$ (see an example in Table 2), which encodes the time structure of the pattern in its rows and the instrumentation in its columns.

**Table 2:** Matrix $S_{13}$ representing the sensory data of the *Bravado* drum pattern, played by Neil Peart (stimulus 13). For a transcription of this pattern, see Fig. 2 (c). The matrix $S_{13}$ consists of 65 rows and 3 columns of zeros and ones.

	Metric position		Instrumental Layer		
		k=	Bass Drum (l=1)	Snare Drum (l=2)	Cymbals (l=3)
Bar 1	0.00	1	**1**	0	**1**
	0.25	2	0	0	**1**
	0.50	3	0	0	**1**
	0.75	4	0	0	**1**
	1.00	5	**1**	**1**	0
	1.25	6	0	0	**1**
	1.50	7	0	0	**1**
	1.75	8	0	0	**1**
	2.00	9	**1**	0	**1**
	2.25	10	0	0	**1**
	2.50	11	0	0	**1**
	2.75	12	0	0	**1**
	3.00	13	**1**	**1**	0
	3.25	14	0	0	**1**
	3.50	15	0	0	**1**
	3.75	16	0	0	**1**
Bar 2	4.00	17	**1**	0	**1**
	4.25	18	0	0	**1**
	4.50	19	0	0	**1**
	4.75	20	0	0	**1**
	5.00	21	**1**	**1**	0
	5.25	22	0	0	**1**
	5.50	23	0	0	**1**
	5.75	24	0	0	**1**
	6.00	25	**1**	0	**1**
	6.25	26	0	0	**1**
	6.50	27	0	0	**1**
	6.75	28	0	0	**1**
	7.00	29	**1**	**1**	0
	7.25	30	0	0	**1**
	7.50	31	0	0	**1**
	7.75	32	0	0	**1**
Bar 3	8.00	33	**1**	0	**1**
	8.25	34	0	0	**1**
	8.50	35	0	0	**1**
	8.75	36	0	0	**1**
	9.00	37	**1**	**1**	0
	9.25	38	0	0	**1**
	9.50	39	0	0	**1**
	9.75	40	0	0	**1**
	10.00	41	**1**	0	**1**
	10.25	42	0	0	**1**
	10.50	43	0	0	**1**
	10.75	44	0	0	**1**
	11.00	45	**1**	**1**	0
	11.25	46	0	0	**1**
	11.50	47	0	0	**1**
	11.75	48	0	0	**1**
Bar 4	12.00	49	**1**	0	**1**
	12.25	50	0	0	**1**
	12.50	51	0	0	**1**
	12.75	52	0	0	**1**
	13.00	53	**1**	**1**	0
	13.25	54	0	0	**1**
	13.50	55	0	0	**1**
	13.75	56	0	0	**1**
	14.00	57	**1**	0	**1**
	14.25	58	0	0	**1**
	14.50	59	0	0	**1**
	14.75	60	0	0	**1**
	15.00	61	**1**	**1**	0
	15.25	62	0	0	**1**
	15.50	63	0	0	**1**
	15.75	64	0	0	**1**
Bar 5	16.00	65	**1**	0	**1**
2. *Deriving the internal model*$M_{0}$*of an enculturated listener from a corpus of drum patterns*: This step generates the internal model (or expectations) of an enculturated listener based on previous experience. It specifies the probability of a note onset at each time point k in each instrumental layer l based on a corpus of drum patterns, and it represents the probabilities in the 65×3 matrix $M_{0}$ (see Table 3).

**Table 3:** Matrix $M_{0}$: Probabilities that an event happens in an instrumental layer on a metric position, based on a corpus of n=211 popular music drum patterns. The probabilities repeat every bar. Events of the generic backbeat pattern in bold print, see also Fig. 2 (a).

	Metric position		Instrumental Layer		
		k=	Bass Drum (l=1)	Snare Drum (l=2)	Cymbals (l=3)
Bar 1	0.00	1	**0.914**	0.039	**0.607**
	0.25	2	0.033	0.102	0.129
	0.50	3	0.320	0.057	**0.642**
	0.75	4	0.192	0.040	0.132
	1.00	5	0.184	**0.887**	**0.719**
	1.25	6	0.063	0.084	0.134
	1.50	7	0.246	0.074	**0.664**
	1.75	8	0.162	0.187	0.156
	2.00	9	**0.641**	0.082	**0.717**
	2.25	10	0.065	0.209	0.143
	2.50	11	0.454	0.084	**0.626**
	2.75	12	0.206	0.056	0.141
	3.00	13	0.191	**0.847**	**0.683**
	3.25	14	0.133	0.085	0.126
	3.50	15	0.238	0.145	**0.623**
	3.75	16	0.108	0.187	0.143
Bar 2	4.00	17	**0.914**	0.039	**0.607**
	4.25	18	0.033	0.102	0.129
	4.50	19	0.320	0.057	**0.642**
	…	…	…	…	…
	…	…	…	…	…
	…	…	…	…	…
Bar 5	16.00	65	**0.914**	0.039	**0.607**
3. *Learning the pattern in a Bayesian process and updating the internal model*$M_{i}$: In this step, a learning process is implemented, which simulates how the listener adapts the internal model as the drum pattern unfolds in time and is being perceived. This process starts with the internal model $M_{0}$ of the enculturated listener. Yet, probabilities for every time point k and for every instrumental layer l are updated in a Bayesian way on the basis of the events in the drum pattern $S_{i}$ previous to k. This leads to the 65×3 matrix $M_{i}$, which represents the internal model as the drum pattern i unfolds and is being learned (see an example of $M_{i}$ in Table 6). In this Bayesian process, $M_{0}$ represents the prior distribution, $S_{i}$ is the new data, and $M_{i}$ is the posterior distribution.
4. *Estimating complexity*: In this step, the value of the surprisal function is estimated on the basis of the sensory data $S_{i}$ and the internal model $M_{i}$ for each time point k and each instrumental layer l. Large discrepancies between $S_{i}$ and $M_{i}$ will result in high surprisal. The surprisal values will be stored in the 65×3 matrix $\Sigma_{i}$, which will allow to calculate the effort $E_{i}$ and the estimated complexity ${\hat{\gamma }}_{i}$ for each stimulus. An example of $\Sigma_{i}$ can be seen in Table 7*.*
5. *Determining the strength*λ*of the prior model*$M_{0}$: In this step, the strength λ of the prior distribution (the enculturated internal model $M_{0}$) is determined such that the coefficient of determination $R^{2}$ between the perceived (${\hat{\beta }}_{i}$) and estimated (${\hat{\gamma }}_{i}$) complexity values is maximised. The parameter λ determines the inertia of $M_{0}$ in light of new data and can be understood as the inverse of the learning speed.

#### 4.1.1 *Encoding the drum pattern matrix*$S_{i}$

**Fig. 2:**
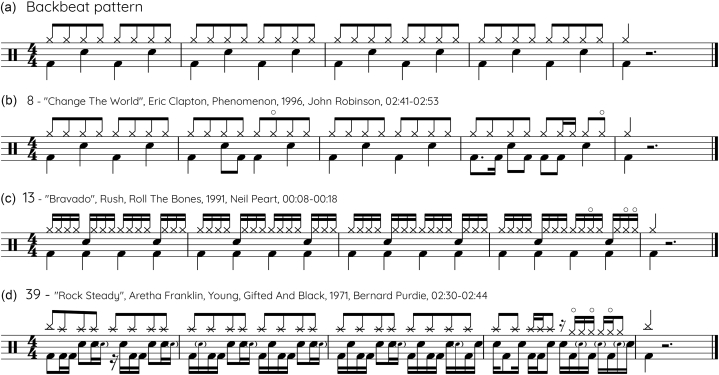
Transcriptions of four drum patterns. (a) Generic backbeat pattern. (b) “Change The World” (stimulus 8). (c) “Bravado” (stimulus 13). (d) “Rock Steady” (stimulus 39). The author would like to thank Florian Hoesl for preparing Fig. 2.

Fig. 2 shows the generic backbeat pattern (a) and three transcribed examples of the 40 drum patterns used in this study (b-d). We understand the time dimension of these 4-bar drum patterns as a grid of 65 sixteenth note positions on which a note onset may happen or not. Each onset is associated with one of three instrumental categories: either the onset is a kick of the bass drum (notated above the bottom line of the staff), or it is a stroke on the snare drum (notated above the middle line), or it is an event involving one of the cymbals (most often notated above the top line). The drum patterns used in this study do not contain events played on any other drum (e.g. toms) or percussion instruments.

The 65×3 drum pattern matrix $S_{i}$ encodes the pattern information for drum pattern i. For an example, see Table 2, which shows the drum pattern matrix $S_{13}$ of the “Bravado” drum pattern, Fig. 2 (c). The K=65 rows of the matrix encode the time information; and the L=3 matrix columns encode the instrument information with bass drum (l=1), snare drum (l=2) and cymbals (l=3). So, if in stimulus i there is an onset at time k in instrument category l, then $S_{i\left(kl\right)}=1$, otherwise $S_{i\left(kl\right)}=0$. Note that the transcribed note length is irrelevant: for an event notated as a quarter note in Fig. 2 (c), worth four 16th notes, only the first 16^th^ note position (where the onset actually happens) is represented as a 1; the remaining three 16^th^ note positions are notated as zeros. The binary encoding in matrix form reduces the drum pattern information substantially relative to the transcription and even more relative to the audio stimulus. Aspects of timbre (e.g. which cymbal is being played, playing techniques), dynamics, microtiming and tempo information are not represented: As outlined in the simplifying assumptions above, we only require the information whether, in drum pattern (i), there is a note onset (1) or not (0) in instrument category (l) at a certain point in time (k).

#### 4.1.2 Deriving the internal model $M_{0}$ of an enculturated listener from a corpus of drum patterns

People with intact hearing who have access to audio-visual media (such as TV, radio, Youtube, etc.) and consume media content on a daily basis are very likely to come into frequent contact with Western popular music, even more so if they live in a Western country. In a majority of popular music songs, the drumset is an essential part of the instrumentation. So, adult listeners are likely to have heard thousands of popular music songs with drum patterns during their previous lifetime. They are likely to have at least an implicit knowledge of how such drum patterns are usually constructed and how they relate to the metric organisation of the music. We define this implicit knowledge as the internal model of a typical drum pattern that an enculturated Western listener has formed in their past life.

We do not have direct access to anybody's past experience or to their particular internal model. However, we can try to approximate the internal model of an enculturated listener by estimating probabilities based on the frequencies encountered in a corpus of drum patterns. In this study, we use 211 drum patterns from the *Lucerne Groove Research Library* as a corpus ([30]). The entire corpus consists of 251 Western popular music drum patterns in 4/4 common time. Forty of these patterns were used to form this study's stimuli set (see also [22]), listed in Table 1. These patterns were excluded from the corpus, and only the remaining 211 patterns were used to estimate the probabilities of the internal model. The 211 patterns consist of 7,196 bass drum kicks, 5,348 snare drum strokes, and 10,912 cymbal strokes. The probability that an event happens in one of the three instrumental layers on any particular metric position within a bar was estimated as the proportion of times the event happens on this metric position in the corpus of 211 patterns.

The proportions are listed in the 65×3 matrix $M_{0}$ (Table 3), whose rows repeat the bar profile of expectations for all 4 bars (plus a fifth repetition of the first downbeat at the very end). On the basis of $M_{0}$, it is very probable that a bass drum event happens on the first downbeat of a pattern ($m_{0\left(1,1\right)}=0.914$), but it is unlikely that the snare drum plays on this position ($m_{0\left(1,2\right)}=0.039$). Yet, a snare drum event is strongly expected on beat two, ($m_{0\left(5,2\right)}=0.887$). We observe in Table 3 that all matrix cells that indicate high probabilities ($m_{0\left(kl\right)}>0.5)$ correspond to events that are compatible with the generic backbeat pattern or “archetypical rock beat” ([35], p. 11, see also Fig. 2 (a)). $M_{0}$ suggests that an enculturated listener expects to hear the boom-tchak pattern of the generic backbeat. $M_{0}$ represents a set of trainable parameters of the model that are empirically estimated from a corpus of drum patterns. These parameters are trainable, because the probabilities would take different values, if they were based (or trained) on a different corpus.

#### 4.1.3 *Learning the pattern as a Bayesian process and updating the internal model*$M_{i}$

According to predictive coding theory ([16]), the organism's overall goal is to stay in healthy conditions. It tries to achieve this by keeping surprisal low and thus avoiding potentially dangerous surprises. The organism may improve the prediction capabilities of the internal model m(t) through a process of learning.

When a drum pattern starts to play, the listener interprets it using their internal model that we approximated in model $M_{0}$, and that is based on previous experience. As the pattern unfolds, the listener gets to know the pattern's specific characteristics and adapts the internal model accordingly. So, listening is a learning process which consists in forming a new model $M_{i}$ that is more appropriate for pattern i than the generic model $M_{0}$. We may understand this learning process as an adaptation of expectations that takes into account the pattern up to time k, the 4/4 time signature and the tendency of many drum patterns to repeat every two quarter notes (8 sixteenths), every bar (16 sixteenths), every 2 bars (32 sixteenths) or 4 bars (64 sixteenths). At every time point k in instrument layer l, the probability of an event will increase when there already were events in this layer at times k−8, k−16, k−32, and/or k−64, and decrease otherwise.

We model this learning experience as a Bayesian process for which the internal model $M_{0}$ serves as the prior, listening to the pattern i (using $S_{i}$ and a method to update the expectancies of the internal model) provides the new data, and the updated internal model $M_{i}$ is the posterior. This process is implemented as a conjugate analysis using beta distributions (see Ref. [36] p. 34ff.; [37], p. 24ff.; [38], p. 49ff.). Conjugacy implies that the prior and posterior distributions will belong to the same distribution family. Beta distributions are a family of probability distributions for real numbers defined on the range [0,1] that can be used in conjugate analysis if the parameter of interest is a proportion or a probability. This is the case here, because we want to update the prior probabilities of $M_{0}$ to the posterior probabilities of $M_{i}$ at the 3×65 positions of a 4-bar drum pattern, consequently a conjugate analysis using beta distributions is possible.

We operationalise the updating process by first defining 65×3 beta distributions such that the modes of these distributions are equal to the 65×3 probabilities of the prior model $M_{0}$. Each beta distribution is governed by two parameters, a and b ([39], p. 480f.). Beta distributions with large a and small b generate random numbers near to 1. Conversely, beta distributions with small a and large b generate random numbers near to 0. In our modelling, we will use the location of the mode of the beta distribution as a point estimate to indicate the probability that an event happens in the drum pattern. The mode m of a beta distribution is given by:

(10) $$m=\frac{a-1}{a+b-2}$$

The strength (or inertia) of the prior distribution is given by:

(11) λ=a+b

When λ takes a large value, then the prior distribution tends to be strong and new data will only have a weak influence on the posterior distribution. Conversely, when the prior distribution is weak (low λ), the new data will strongly affect the posterior distribution. The optimal value for parameter λ will be estimated at a later moment in this study in an optimisation process.

We can substitute equation (11), (10) and solve (10) for a and b obtaining:

a=(λ−2)m+1

b=λ−(λ−2)m−1

Expanding these formulas to calculate all 65×3 a and b values corresponding to the prior model $M_{0}$ simultaneously using matrix operations yields:

$$A_{0}=\left(\lambda -2\right)M_{0}+J$$

$$B_{0}=\lambda J-\left(\lambda -2\right)M_{0}-J$$

where $A_{0}$ and $B_{0}$ are 65×3 matrices that collect the a and b values and J is the 65×3 all-ones matrix. With this, we have defined all 65×3 prior beta distributions that have their modes at $M_{0}$ and prior strength λ, see Table 4, Table 5.

**Table 4:** Matrix $A_{0}$: Parameters $a_{0\left(kl\right)}$ of the 65×3 beta distributions (*strength of prior distribution*λ=3.56) that have their modes at $M_{0}$. The values repeat every bar.

	Metric position		Instrumental Layer		
		k=	Bass Drum (l=1)	Snare Drum (l=2)	Cymbals (l=3)
Bar 1	0.00	1	2.426	1.061	1.947
	0.25	2	1.051	1.159	1.201
	0.50	3	1.500	1.090	2.001
	0.75	4	1.299	1.062	1.205
	1.00	5	1.287	2.384	2.122
	1.25	6	1.098	1.131	1.210
	1.50	7	1.384	1.116	2.035
	1.75	8	1.252	1.291	1.244
	2.00	9	2.000	1.128	2.119
	2.25	10	1.101	1.325	1.224
	2.50	11	1.708	1.130	1.977
	2.75	12	1.321	1.087	1.220
	3.00	13	1.299	2.322	2.066
	3.25	14	1.207	1.132	1.196
	3.50	15	1.371	1.226	1.971
	3.75	16	1.169	1.291	1.223
Bar 2	4.00	17	2.426	1.061	1.947
	4.25	18	1.051	1.159	1.201
	4.50	19	1.500	1.090	2.001
	…	…	…	…	…
	…	…	…	…	…
	…	…	…	…	…
Bar 5	16.00	65	2.426	1.061	1.947

**Table 5:** Matrix $B_{0}$: Parameters $b_{0\left(kl\right)}$ of the 65×3 beta distributions (*strength of prior distribution*λ=3.56) that have their modes at $M_{0}$. The values repeat every bar. Note that for all k and l it is true that $a_{0\left(kl\right)}+b_{0\left(kl\right)}=\lambda =3.56$, see equation (11).

	Metric position		Instrumental Layer		
		k=	Bass Drum (l=1)	Snare Drum (l=2)	Cymbals (l=3)
Bar 1	0.00	1	1.134	2.499	1.613
	0.25	2	2.509	2.401	2.359
	0.50	3	2.060	2.470	1.559
	0.75	4	2.261	2.498	2.355
	1.00	5	2.273	1.176	1.438
	1.25	6	2.462	2.429	2.350
	1.50	7	2.176	2.444	1.525
	1.75	8	2.308	2.269	2.316
	2.00	9	1.560	2.432	1.441
	2.25	10	2.459	2.235	2.336
	2.50	11	1.852	2.430	1.583
	2.75	12	2.239	2.473	2.340
	3.00	13	2.261	1.238	1.494
	3.25	14	2.353	2.428	2.364
	3.50	15	2.189	2.334	1.589
	3.75	16	2.391	2.269	2.337
Bar 2	4.00	17	1.134	2.499	1.613
	4.25	18	2.509	2.401	2.359
	4.50	19	2.060	2.470	1.559
	…	…	…	…	…
	…	…	…	…	…
	…	…	…	…	…
Bar 5	16.00	65	1.134	2.499	1.613

If an event happens in instrument l at metric position k, then the probabilities of an event happening half a bar later (k+8), 1 bar later (k+16), 2 bars later (k+32) or 4 bars later (k+64) need to be increased. This can be achieved by increasing the parameters a at these future positions (which implies that the mode of the beta distribution and thus the probability of an event increases). Conversely, if there is a rest at position k, then the future probabilities should be reduced, which can be achieved by increasing the parameters b at these future positions (decreasing the mode of the beta distribution and thus the probability of an event).

To this end, we first define the update matrix U, which is a 65×65 matrix of 4085 zeros and 140 ones. It takes care of projecting the expectations half a bar, 1 bar, 2 bars, or 4 bars into the future. Matrix U is provided in Fig. 6. For all ones in U, the column number indicates the position in time that affects another position, and the row number indicates the position that is being affected. To make an example: the cell $u_{9,1}$ takes value 1, and this means that what happens at k=1 (column number) affects what happens at k=9 (row number), which is half a bar later. U can be understood as a hyper-parameter of the model, because it encapsulates listeners’ knowledge of the 4/4 m and their experience that patterns tend to repeat with periods of half a bar, 1 bar, etc.

When a stroke is played at metric position k in instrument l, then the probabilities of the corresponding future events need to increase. This is equivalent to increasing the corresponding a parameters of the beta distributions by one and is achieved carrying out the following operation:

(12) $$A_{i}=US_{i}+A_{0}\text{,}$$

where $A_{i}$ is the matrix of the coefficients a for stimulus i after learning has taken place, and U, $S_{i}$, and $A_{0}$ are defined as above.

When there is no stroke at metric position k in instrument l, then the probability of the corresponding future events should be decreased. This is equivalent to increasing the b parameters of the beta distributions by one and is achieved by the following matrix operation:

(13) $$B_{i}=U\left(J-S_{i}\right)+B_{0}\text{,}$$

where $B_{i}$ is the matrix of the coefficients b for stimulus i after learning has taken place. U, J, $S_{i}$ and $B_{0}$ are defined as above. The operation $J-S_{i}$ swaps zeros and ones of the pattern matrix such that all onsets are represented as zeros and all rests as ones. This ensures that the rests are projected forward to the b parameters.

The updating process operates similar to the conjugate Bayesian analysis for a binomial proportion with the beta distributions specified by parameters $A_{0}$ and $B_{0}$ acting as prior distributions. Those specified by $A_{i}$ and $B_{i}$ are the posterior distributions. The only difference is that the counts that contribute to the $A_{i}$ and $B_{i}$ are not observed successes or failures in Bernoulli experiments, but counts of the times a future metric position is made more or less probable based on previous events.

We finally use the parameters $A_{i}$ and $B_{i}$ of the posterior beta distributions to calculate the modes of the model $M_{i}$, which are point estimates of the probabilities of the internal model after learning stimulus i has taken place. This is achieved using equation (10), which in matrix form becomes:

$$M_{i}=\left(A_{i}-1\right)⊘\left(A_{i}+B_{i}-2\right),$$

where ⊘ indicates the Hadamard division (element-wise division of two matrices of the same dimension, see Ref. [40]). The probabilities of the updated internal model $M_{13}$ after learning Brad Wilk's pattern of “Bravado” can be studied in Table 6.

**Table 6:** Model $M_{13}$ for the *Bravado* pattern, played by Neil Peart (stimulus 13), after learning has taken place. For a transcription of this pattern, see Fig. 2 (c). Probabilities >0.5 are in bold print.

	Metric position		Instrumental Layer		
		k=	Bass Drum (l=1)	Snare Drum (l=2)	Cymbals (l=3)
Bar 1	0.00	1	**0.914**	0.039	**0.607**
	0.25	2	0.033	0.102	0.129
	0.50	3	0.320	0.057	**0.642**
	0.75	4	0.192	0.040	0.132
	1.00	5	0.184	**0.887**	**0.719**
	1.25	6	0.063	0.084	0.134
	1.50	7	0.246	0.074	**0.664**
	1.75	8	0.162	0.187	0.156
	2.00	9	**0.781**	0.050	**0.828**
	2.25	10	0.039	0.127	0.478
	2.50	11	0.277	0.051	**0.772**
	2.75	12	0.125	0.034	0.477
	3.00	13	**0.507**	**0.907**	0.416
	3.25	14	0.081	0.052	0.467
	3.50	15	0.145	0.088	**0.770**
	3.75	16	0.066	0.114	0.478
Bar 2	4.00	17	**0.962**	0.017	**0.828**
	4.25	18	0.014	0.045	**0.618**
	4.50	19	0.140	0.025	**0.843**
	4.75	20	0.084	0.017	**0.619**
	5.00	21	**0.643**	**0.951**	0.315
	5.25	22	0.028	0.037	**0.621**
	5.50	23	0.108	0.032	**0.853**
	5.75	24	0.071	0.082	**0.630**
	6.00	25	**0.843**	0.036	**0.876**
	6.25	26	0.028	0.091	**0.625**
	6.50	27	0.199	0.037	**0.836**
	6.75	28	0.090	0.024	**0.624**
	7.00	29	**0.646**	**0.933**	0.299
	7.25	30	0.058	0.037	**0.617**
	7.50	31	0.104	0.064	**0.835**
	7.75	32	0.048	0.082	**0.624**
Bar 3	8.00	33	**0.971**	0.013	**0.866**
	8.25	34	0.011	0.035	**0.702**
	8.50	35	0.110	0.020	**0.877**
	8.75	36	0.066	0.014	**0.703**
	9.00	37	**0.721**	**0.961**	0.246
	9.25	38	0.021	0.029	**0.704**
	9.50	39	0.084	0.025	**0.885**
	9.75	40	0.055	0.064	**0.711**
	10.00	41	**0.877**	0.028	**0.903**
	10.25	42	0.022	0.071	**0.707**
	10.50	43	0.155	0.029	**0.872**
	10.75	44	0.070	0.019	**0.706**
	11.00	45	**0.723**	**0.948**	0.234
	11.25	46	0.045	0.029	**0.701**
	11.50	47	0.081	0.050	**0.871**
	11.75	48	0.037	0.064	**0.707**
Bar 4	12.00	49	**0.971**	0.013	**0.866**
	12.25	50	0.011	0.035	**0.702**
	12.50	51	0.110	0.020	**0.877**
	12.75	52	0.066	0.014	**0.703**
	13.00	53	**0.721**	**0.961**	0.246
	13.25	54	0.021	0.029	**0.704**
	13.50	55	0.084	0.025	**0.885**
	13.75	56	0.055	0.064	**0.711**
	14.00	57	**0.877**	0.028	**0.903**
	14.25	58	0.022	0.071	**0.707**
	14.50	59	0.155	0.029	**0.872**
	14.75	60	0.070	0.019	**0.706**
	15.00	61	**0.723**	**0.948**	0.234
	15.25	62	0.045	0.029	**0.701**
	15.50	63	0.081	0.050	**0.871**
	15.75	64	0.037	0.064	**0.707**
Bar 5	16.00	65	**0.976**	0.011	**0.890**

It may be confusing that the matrix operations of equations (12), (13) update the coefficients $A_{i}$ and $B_{i}$ at once, without reference to the temporality involved (the fact that only onsets at time k−8, k−16 etc. affect the probability of an onset at time k). The temporal aspect is encoded in the update matrix U (Fig. 6) which is of lower triangular form (all ones are below the main diagonal). This makes sure that probabilities at every point in time are only affected by the past. This can be verified by comparing $M_{0}$ (Table 3) and $M_{13}$ (Table 6): you may note that, for the first eight sixteenth notes (k=1 through k=8), the two tables are identical, because it needs a temporal distance of at least eight sixteenth notes (or half a bar) until the past affects the present, until learning takes place and the probabilities of matrix $M_{13}$ change.

#### 4.1.4 Estimating complexity

With the probabilities of the internal model $M_{i}$ we can now proceed to calculate the surprisal experienced by an enculturated listener who is learning the pattern as it unfolds. Surprisal, as defined in equation (3), is operationalised as follows:

$$\Sigma_{i}=-\log\left({|S}_{i}+M_{i}-J|\right)$$

where the surprisal matrix $\Sigma_{i}$ is a 65×3 matrix that represents the estimated surprisal for all K=65 discrete time positions in the L=3 instrumental layers. As defined above, $S_{i}$ is the matrix representing the sensory data relating to pattern i in binary form; $M_{i}$ is the internal model as a result of short-term learning, J is the 65×3 all-ones matrix, and the vertical bars are the absolute operator (see the surprisal matrix $\Sigma_{13}$ of the “Bravado” pattern in Table 7).

**Table 7:** Surprisal matrix $\Sigma_{13}$ for the *Bravado* pattern, played by Neil Peart (stimulus 13). For a transcription of this pattern, see Fig. 2 (c). Surprisals >0.5 are in bold print.

	Metric position		Instrumental Layer		
		k=	Bass Drum (l=1)	Snare Drum (l=2)	Cymbals (l=3)
Bar 1	0.00	1	0.090	0.040	0.499
	0.25	2	0.033	0.107	**2.047**
	0.50	3	0.386	0.059	0.444
	0.75	4	0.213	0.041	**2.029**
	1.00	5	**1.692**	0.119	**1.270**
	1.25	6	0.065	0.088	**2.006**
	1.50	7	0.282	0.077	0.410
	1.75	8	0.176	0.207	**1.855**
	2.00	9	0.247	0.051	0.189
	2.25	10	0.040	0.136	**0.738**
	2.50	11	0.324	0.052	0.259
	2.75	12	0.134	0.035	**0.741**
	3.00	13	**0.679**	0.098	**0.538**
	3.25	14	0.084	0.053	**0.761**
	3.50	15	0.156	0.093	0.261
	3.75	16	0.068	0.121	**0.739**
Bar 2	4.00	17	0.038	0.017	0.189
	4.25	18	0.014	0.046	0.481
	4.50	19	0.151	0.026	0.171
	4.75	20	0.088	0.018	0.479
	5.00	21	0.442	0.051	0.379
	5.25	22	0.028	0.038	0.477
	5.50	23	0.114	0.033	0.160
	5.75	24	0.074	0.085	0.462
	6.00	25	0.171	0.036	0.132
	6.25	26	0.029	0.096	0.471
	6.50	27	0.222	0.037	0.179
	6.75	28	0.094	0.025	0.472
	7.00	29	0.438	0.069	0.356
	7.25	30	0.060	0.038	0.483
	7.50	31	0.110	0.066	0.181
	7.75	32	0.049	0.085	0.471
Bar 3	8.00	33	0.030	0.013	0.144
	8.25	34	0.011	0.035	0.354
	8.50	35	0.116	0.020	0.131
	8.75	36	0.068	0.014	0.353
	9.00	37	0.327	0.039	0.282
	9.25	38	0.022	0.029	0.351
	9.50	39	0.088	0.026	0.122
	9.75	40	0.057	0.066	0.341
	10.00	41	0.131	0.028	0.102
	10.25	42	0.022	0.074	0.347
	10.50	43	0.169	0.029	0.137
	10.75	44	0.073	0.019	0.348
	11.00	45	0.324	0.054	0.266
	11.25	46	0.046	0.029	0.355
	11.50	47	0.085	0.051	0.138
	11.75	48	0.038	0.066	0.347
Bar 4	12.00	49	0.030	0.013	0.144
	12.25	50	0.011	0.035	0.354
	12.50	51	0.116	0.020	0.131
	12.75	52	0.068	0.014	0.353
	13.00	53	0.327	0.039	0.282
	13.25	54	0.022	0.029	0.351
	13.50	55	0.088	0.026	0.122
	13.75	56	0.057	0.066	0.341
	14.00	57	0.131	0.028	0.102
	14.25	58	0.022	0.074	0.347
	14.50	59	0.169	0.029	0.137
	14.75	60	0.073	0.019	0.348
	15.00	61	0.324	0.054	0.266
	15.25	62	0.046	0.029	0.355
	15.50	63	0.085	0.051	0.138
	15.75	64	0.038	0.066	0.347
Bar 5	16.00	65	0.024	0.011	0.117

This operationalisation of surprisal is symmetric, meaning that the presence of an unexpected event is as surprising as the absence of an expected event. Let us for example assume that an event happens at time k in instrument l, then $S_{i\left(kl\right)}=1$, and let us also assume that this event was highly expected by the internal model, say $M_{i\left(kl\right)}=0.9$, then we get:

$$\Sigma_{i\left(kl\right)}=-\log\left({|S}_{i\left(kl\right)}+M_{i\left(kl\right)}-J_{\left(kl\right)}|\right)=-\log\left(\left|1+0.9-1\right|\right)=-\log\left(0.9\right)≅0.105.$$

Conversely, if there is no event on position k and l ($S_{i\left(kl\right)}=0$), and an event was improbable to happen according to the internal model ($M_{i\left(kl\right)}=0.1$), then we get:

$$\Sigma_{i\left(kl\right)}=-\log\left({|S}_{i\left(kl\right)}+M_{i\left(kl\right)}-J_{\left(kl\right)}|\right)=-\log\left(\left|0+0.1-1\right|\right)=-\log\left(0.9\right)≅0.105\text{.}$$

This exemplifies the symmetry of estimating surprisal from events and non-events. Further, the calculations for the snare drum, bass drum and cymbals are carried out identically. This means that surprises in the three instrumental layers have the same weight.

We estimate the effort $E_{i}$ of learning stimulus i as the grand sum of all values of the surprisal matrix $\Sigma_{i}$ in analogy to equation (4):

$$E_{i}=\sum_{k=1}^{65}\sum_{l=1}^{3}\Sigma_{i\left(kl\right)}\text{.}$$

This leads to the effort estimates $E_{i}$ for each of the forty patterns listed in Table 8.

**Table 8:** *Perceived complexity* (Bradley-Terry estimates ${\hat{\beta }}_{i}$), estimated effort ($E_{i}$), its natural logarithm (log($E_{i})$), and the *estimated complexity* (${\hat{\gamma }}_{i}=C\;\log\left(E_{i}\right)+D$, where C=2.492 and D=−6.927).

Stimulus i	Song Title	Perceived Complexity ${\hat{\beta }}_{i}$	Effort $E_{i}$	Log Effort $\log\left(E_{i}\right)$	Estimated Complexity ${\hat{\gamma }}_{i}$
1	A Kind Of Magic	0.400	21.580	3.072	0.728
2	(Sittin’ On) The Dock Of The Bay	0.408	20.934	3.041	0.653
3	Smells Like Teen Spirit	0.476	19.253	2.958	0.444
4	Boogie Wonderland	0.573	23.560	3.160	0.947
5	Vultures	0.784	20.583	3.024	0.611
6	Kashmir	1.182	24.532	3.200	1.048
7	Street Of Dreams	1.210	23.766	3.168	0.969
8	Change The World	1.230	29.535	3.386	1.511
9	Let's Dance	1.263	30.397	3.414	1.582
10	Space Cowboy	1.415	28.527	3.351	1.424
11	I Feel For You	1.632	33.863	3.522	1.851
12	Virtual Insanity	1.859	32.447	3.480	1.745
13	Bravado	1.902	42.047	3.739	2.391
14	Let's Go Dancing	1.951	35.809	3.578	1.991
15	Discipline	2.091	32.442	3.479	1.744
16	Pass The Peas	2.151	30.343	3.413	1.578
17	The Pump	2.189	49.724	3.906	2.809
18	Roxanne	2.216	32.386	3.478	1.740
19	Dreamin’	2.413	33.715	3.518	1.840
20	Soon I'll Be Loving You Again	2.511	40.282	3.696	2.284
21	Summer Madness	2.530	47.189	3.854	2.678
22	Listen Up!	2.586	56.968	4.042	3.148
23	Jungle Man	2.752	59.883	4.092	3.272
24	Shake Everything You Got	3.051	56.440	4.033	3.124
25	Chicken	3.052	67.975	4.219	3.588
26	Cissy Strut	3.080	62.438	4.134	3.376
27	Far Cry	3.120	59.284	4.082	3.247
28	Alone + Easy Target	3.130	41.472	3.725	2.356
29	Soul Man	3.263	70.562	4.256	3.681
30	Ain't Nobody	3.300	62.312	4.132	3.371
31	Diggin' On James Brown	3.314	53.148	3.973	2.975
32	In The Stone	3.342	62.234	4.131	3.368
33	Southwick	3.360	70.860	4.261	3.691
34	You Can Make It If You Try	3.447	58.893	4.076	3.230
35	The Dump	3.464	79.680	4.378	3.984
36	Killing In The Name Of	3.564	38.898	3.661	2.197
37	Cold Sweat	3.763	50.023	3.912	2.824
38	Hyperpower	3.902	81.091	4.396	4.027
39	Rock Steady	4.394	102.850	4.633	4.620
40	Jelly Belly	4.701	91.439	4.516	4.327

Next, we need to estimate the constants C and D, and calculate the *estimated complexity* values ${\hat{\gamma }}_{i}$. We intend that the ${\hat{\gamma }}_{i}$ have the same mean and standard deviation as the empirical *perceived complexity* values ${\hat{\beta }}_{i}$ that were estimated on the basis of the listening experiment in Senn et al. ([22]). This will make the $\hat{\gamma }$ s and $\hat{\beta }$ s comparable, not only in terms of their location and spread, but also in their probabilistic interpretation specified by the Bradley-Terry model. Using information from Table 8 we obtain:

$$C=\frac{SD\left[\beta \right]}{SD\left[\log\left(E\right)\right]}=2.492082≅2.492\text{,}$$

$$D=mean\left[\beta \right]-mean\left[\log\left(E\right)\right]\times \frac{SD\left[\beta \right]}{SD\left[\log\left(E\right)\right]}=-6.926637≅-6.927\text{,}$$

where β is the vector of the forty $\beta_{i}$ values, and E is the vector of the forty $E_{i}$ values shown in Table 8. Substituting the values for C and D into equation (6), we obtain the following formula for the *estimated complexity* ${\hat{\gamma }}_{i}$ of stimulus i:

$${\hat{\gamma }}_{i}=C\;\log\left(E_{i}\right)+D=2.492\times \log\left(E_{i}\right)-6.927\text{.}$$

**Fig. 3:**
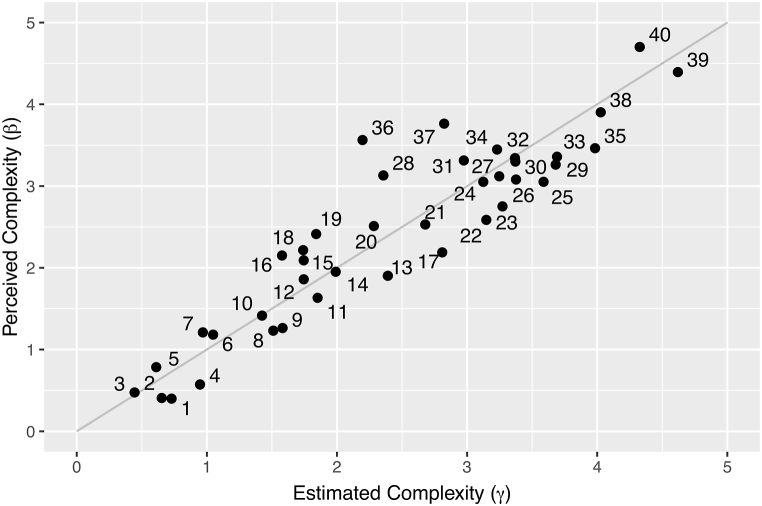
Scatterplot of *estimated complexity* (${\hat{\gamma }}_{i}$) on the x-axis and the empirically measured *perceived complexity* (${\hat{\beta }}_{i}$) on the y-axis. These values are listed in Table 1, Table 8.

The resulting ${\hat{\gamma }}_{i}$ are presented in Table 1 (and in Table 8). Fig. 3 shows a scatterplot of the *estimated complexity* values ${\hat{\gamma }}_{i}$ (on the horizontal axis) against the *perceived complexity* measures ${\hat{\beta }}_{i}$ (on the vertical axis). The gray diagonal line marks $\hat{\gamma }=\hat{\beta }$, the locations where *estimated complexity* agrees exactly with the *perceived complexity* values. For points above this diagonal, the *perceived complexity* of the stimulus is greater than estimated by the model. Conversely, for points below the diagonal, *perceived complexity* is less than estimated. Note that the ${\hat{\gamma }}_{i}$ are just a linear transformation of $\log\left(E_{i}\right)$. This means that parameters C and D do not affect the relationship between *estimated* and *perceived complexity* (as measured by the Pearson correlation r or the coefficient of determination $R^{2}$) and are therefore not parameters changing the fit of the model.

#### 4.1.5 *Estimating the strength*λ*of the prior model*$M_{0}$

As a last step, we need to clarify how the optimal strength λ of the prior distributions was determined. In the context of this model, λ can be understood as a hyper-parameter that is an inverse measure of learning speed. We want to choose λ such that the ${\hat{\gamma }}_{i}$ explain as much of the variance of the ${\hat{\beta }}_{i}$ as possible. The proportion of explained variance is operationalised as the *coefficient of determination* ($R^{2}$) between ${\hat{\beta }}_{i}$ and ${\hat{\gamma }}_{i}$. If we optimise λ on the entire set of 40 drum patterns, we run the risk of overfitting the data, because the hyper-parameter λ and the outcome criterion $R^{2}$ are estimated on the same sample of forty patterns. In order to minimise overfitting, the following simulation approach was chosen. In each of n=10000 iterations:

1. The 40 drum patterns were randomly split into a training and a test set, each with 20 drum patterns.
2. An optimal λ was estimated using the training set. This optimisation consisted in varying the strength of the prior between λ=2.1 and λ=10.0 in steps of 0.1, calculating the coefficient of determination $R^{2}$ with respect to the 20 training set patterns for each value of λ, and selecting the optimal value of λ that maximises $R^{2}$.
3. This optimal λ was then used to estimate $R^{2}$ with respect to the 20 test set patterns.

**Fig. 4:**
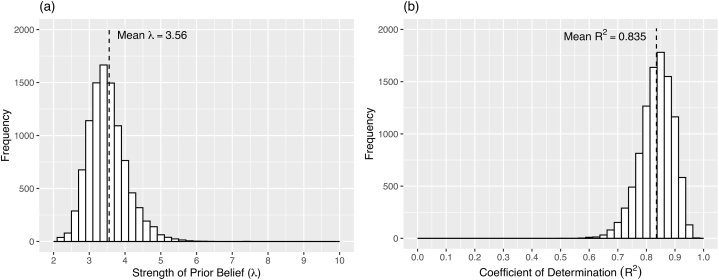
Histograms: (a) Values of the strength of the prior (λ), calculated on the 10,000 simulated training sets. The mean was at λ=3.56 and a 95% prediction interval spanned (2.7, 4.8). (b) Values of the model fit ($R^{2}$) between the empirical ${\hat{\beta }}_{i}$ and the estimated ${\hat{\gamma }}_{i}$, calculated on the 10,000 test sets. The mean model fit was $R^{2}=.835$.

Fig. 4 shows histograms of the 10,000 optimal λ (resulting from step 2 of the simulation) and $R^{2}$ values (resulting from step 3). The optimal lambdas (a) had a mean of λ=3.56 and a range of [2.1,7.4]. The empirical distribution of λ had its .025-quantile at 2.7 and its .975-quantile at 4.8; consequently a prediction interval that contains the optimal λ in 95% of the cases can be estimated to span (2.7, 4.8). The simulated $R^{2}$ values (b) had a mean of .835, a range of (.510, .968), and a .01-quantile of .677. So, in 99% of the cases $R^{2}\geq .677$.

For practical reasons, it is useful to provide a heuristic λ value for future complexity estimations. A good candidate is the mean optimal lambda value of λ=3.56. In order to estimate the fit of the model when the strength of the prior is fixed at λ=3.56, a second simulation was run with n=10000 new test sets of 20 drum patterns, randomly drawn from the 40 patterns. For each of these simulated test sets, $R^{2}$ was calculated using λ=3.56. The resulting empirical distribution of $R^{2}$ values had a mean of .849 with a range of (.471, .967), and a .01-quantile of .709. Future applications of the model are likely to show a fit with a similar distribution: we can expect that the model predicts *perceived complexity* with a fit of $R^{2}\geq .709$ in 99% of new samples drawn from the same population as those used in this study (Western popular music drum patterns, 4 bars duration, 4/4 time signature, instrumentation with bass drum, snare drum, and cymbals). The *estimated complexity* (${\hat{\gamma }}_{i}$) values for this study's forty drum patterns (see Fig. 3, Table 1, Table 8) were calculated using the mean strength of prior belief of λ=3.56 and achieved a model fit with the empirical *perceived complexity* (${\hat{\beta }}_{i}$) values of $R^{2}=0.8517591≅0.852$.

## 5 Discussion

This study presented a model of perceived popular music drum pattern complexity, based on ideas from predictive coding (PC). In particular, the model claimed that the perceived complexity of a drum pattern depended on the surprisal experienced by the listener. The model's complexity estimations turned out to be very good: with a fit of $R^{2}=.852$, the model outperformed the two *Indices of Syncopation* by Witek et al. ([9], $R^{2}=.491$) and Hoesl and Senn ([23], $R^{2}=.560$) as a measure of perceived complexity by a wide margin (for estimations of fit, see Ref. [22], p. 18). The new method only required the optimisation of one single parameter (namely the prior model strength λ) to achieve a very good fit (note that C and D were not optimised, but calculated directly from the means and standard deviations of the ${\hat{\beta }}_{i}$ and ${\log(E}_{i})$ values, and they are irrelevant for the fit of the model). Further, the estimated complexity values ${\hat{\gamma }}_{i}$ are consistent with the empirical Bradley-Terry complexity measures ${\hat{\beta }}_{i}$, which means they have the same straightforward probabilistic interpretation.

In defense of the earlier *Indices of Syncopation* measures it must be acknowleged that they were not specifically designed to reproduce the Bradley-Terry estimates (${\hat{\beta }}_{i}$), and they do not consider the cymbals for their syncopation/complexity calculations. The cymbal layer is quite important explaining *perceived complexity* (${\hat{\beta }}_{i}$): if we omit the contribution of the cymbal layer and estimate complexity only based on the effort associated with the bass drum and the snare drum, the fit of the model drops from $R^{2}=.852$ to $R^{2}=.714$. This implies that $R^{2}=.138$ are uniquely explained by the effort associated with the cymbals layer.

Based on the favourable properties of the model, it may serve as a practical method to reliably estimate the perceived complexity of popular music drum patterns. A dedicated *R* script that provides the complexity estimate of a drum pattern based on its input pattern matrix S is available (see *Data availability statement*). In 99% of drum pattern sets drawn from the repertoire, the *estimated complexity*(${\hat{\gamma }}_{i}$) values can be expected to be good approximations ($R^{2}\geq .709$) of perceived complexity.

### 5.1 Types of drum pattern complexity

**Fig. 5:**
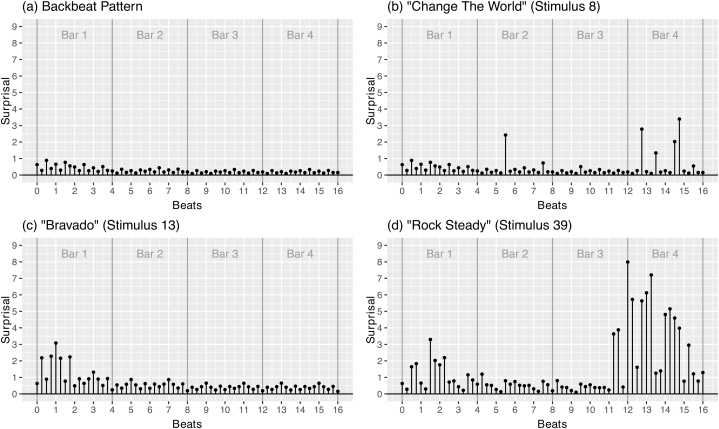
Surprisal plots for four drum patterns. (a) Backbeat pattern. (b) “Change The World” (stimulus 8). (c) “Bravado” (stimulus 13). (d) “Rock Steady” (stimulus 39). Each value of the surprisal function represents one row sum of the surprisal matrix, which is the sum of the surprisal for all three instrumental layers combined at one point in time.

In addition to estimating the overall complexity of a drum pattern, the surprisal function may also serve to describe how drum patterns can be complex in different ways. Fig. 2 shows transcriptions of four drum patterns: the first in Fig. 2 (a) is the generic backbeat pattern, and the three others in Fig. 2 (b–d) are examples from the Senn et al. ([22]) stimuli set. Fig. 5 shows the corresponding surprisal plots, where each value represents the sum of the surprisals caused by the three instrumental layers at each of the 65 discrete time points (the horizontal time axes in Fig. 5 refer to beats measured in quarter notes).

− Fig. 2 (a) is the transcription of the backbeat pattern, which is the most generic pattern in the Western popular music repertoire. The corresponding surprisal plot of Fig. 5 (a) shows only low surprisal values, which diminish as the pattern is being perceived. The overall estimated complexity of this pattern is low ($\hat{\gamma }=0.248$). This agrees with intuition: firstly, the backbeat pattern is what listeners expect to hear (see probabilities of the prior model $M_{0}$ from Table 3). Secondly, since the pattern repeats literally with a lag of k=8 discrete time units (half bars), the prediction by the inner model gets better as the pattern unfolds, leading to a further decline of the surprisal function.
− Stimulus 8 (Fig. 2 (b), “Change the World”) is essentially a backbeat pattern with a few surprising extra notes. Particularly, drummer John Robinson added bass drum notes in bars 2 and 4, and one additional hi-hat (cymbal) note in bar 4. Fig. 5 (b) clearly marks these events as surprises, as discrete time moments where surprisal is high. These are local surprises in an otherwise generic pattern, which increase the estimated complexity of this pattern (${\hat{\gamma }}_{8}=1.511$, see Table 1) compared to the backbeat pattern.
− In stimulus 13 (Fig. 2 (c), “Bravado”), the complexity is not triggered by local events. Rather, this drum pattern by Neil Peart has a more complicated basic pattern: a dense hi-hat voice with events on most 16th note positions (whereas the backbeat pattern is based on 8th notes in the hi-hat). Also, the bass drum plays a four-to-the-floor pattern with kicks on every quarter note (compared to only beats one and three in the backbeat pattern). The unexpected notes of this basic pattern increase the surprisal function values, particularly in the first bar, as seen in Fig. 5 (c). Yet, since the pattern is strictly repeating with a period of half a bar, the model $M_{13}$ learns the features of the pattern and the surprisal function declines. This results in an estimated complexity of ${\hat{\gamma }}_{13}=2.391$.
− Finally, stimulus 39 (Fig. 2 (d), “Rock Steady”) combines both types of complexity: on one hand, drummer Bernard Purdie plays a basic pattern with elaborate bass drum and snare drum voices that deviate from the generic backbeat. This drives surprisal values up in the first bar of Fig. 5 (d), but the model $M_{39}$ learns to expect this (more or less) repeating pattern in bars 2 and 3, and the surprisal values decline. By the end of bar 3, however, the established pattern is changed, which leads to very high surprisal values. The highest value is measured on the first beat of bar 4, where Purdie plays the snare drum (which was very unexpected) instead of the bass drum (which was highly expected). The combined effects of playing an intricate yet repeating basic pattern and adding substantial local surprises in bars 3 and 4 drive estimated complexity up (${\hat{\gamma }}_{39}=4.620$).

**Fig. 6:**
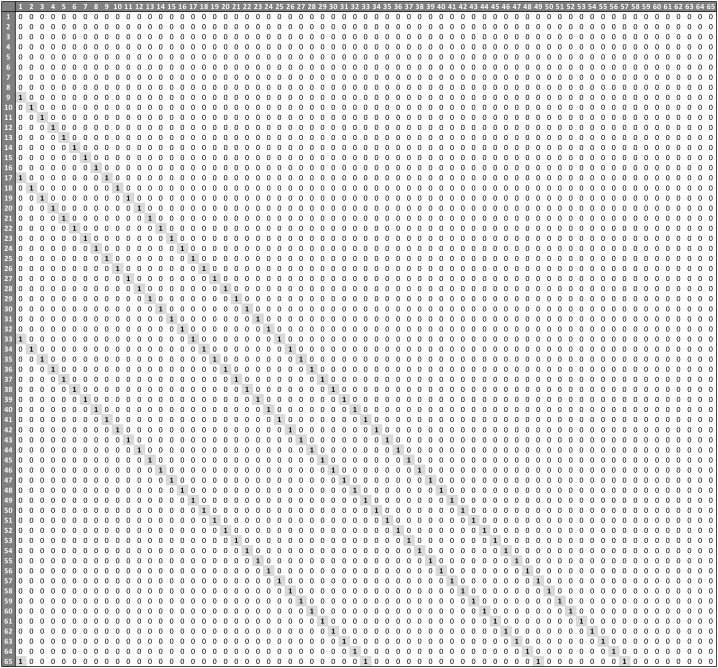
Update matrix U with ones highlighted (bold, gray background).

The complexities of all forty drum patterns can to a certain extent be explained as a combination of the complicatedness (or unconventionality) of a basic repeating pattern, which deviates from the generic backbeat and thus from $M_{0}$, and local surprises (or the omission of repetition), which reduce the learnability of the pattern.

### 5.2 Limitations

The model development started from a variety of simplifying assumptions, which affect the realism of the model and thus can be understood as limitations of the model:

− The decision to use a discrete time domain with K=65 fixed time positions, where an event does or does not happen disregards the influence of tempo, microtiming, and dynamics on perceived complexity. It may well be that listeners perceive a pattern as more complex if it is played faster (or slower), or if some notes are played louder (or softer) or a few milliseconds later (or earlier). Yet, this study's model will not detect such differences.
− The model assumes that the listener is perfectly aware of meter and tempo from the very first moment when a pattern starts to play. Within the model, the sensory data $S_{i}$ is automatically mapped onto the enculturated model $M_{0}$. Yet, in most everyday listening situations, meter and tempo need first to be inferred by the listener as the pattern unfolds, and this might incur additional effort (and thus complexity) on the part of the listener (see also [41], on beat and meter perception). This additional effort is not represented in the model.
− The model treats the three instrumental voices (bass drum, snare drum, cymbals) equally when it comes to their contribution to the surprisal function. Yet, surprising events in one instrument might have a stronger effect on the listener than events in another instrument (see also [9], Supporting Material Text S2, p. 1). In order to explore this possibility, additional weight parameters were added to the model and optimised in order to evaluate the contributions of the instruments. However, the added parameters led to a negligible improvement of model fit ($R^{2}=.854$) and were discarded.
− The model does not consider the properties of short-time memory: an event (or no-event) at time k affects the probabilities of the events at k+8, k+16, k+32, and k+64 in exactly the same way, without regard to limited detention times in short-term memory ([42]). Listeners might remember events that happened half a bar ago more vividly than events that happened 4 bars ago.
− The model assumes an understanding of drum pattern complexity that is compatible with the above definitions of *perceived complexity* and *estimated complexity* relating to pairwise comparison trials and the Bradley-Terry probability model. The model will potentially have a worse fit with perceived complexity measures that have been obtained using different measurement methods (e.g. Likert rating).

Further, there are two major aspects that limit a generalised use of the model: firstly, the modelling work is based on only 40 drum patterns. It is difficult to assess how the potential idiosyncrasies of the selected patterns affect the model parameters and hence the ability of the model to predict the perceived complexity of drum patterns randomly drawn from the repertoire. It would be beneficial to replicate this study based on a larger sample of drum patterns. Secondly, the model can only be applied in a limited number of cases, due to the simplifying assumptions and the specific nature of the empirical data used to fit the model. The model allows to estimate perceived complexity of popular music drum patterns of 4 bars duration (plus beat one of the fifth bar), in common 4/4 time, with binary subdivisions on all levels down to the 16^th^ note level, and with an instrumentation of bass drum, snare drum, and cymbals. The model also assumes that the listener has previous knowledge of popular music drum patterns ($M_{0}$) and is aware of the meter and of the tendency of patterns to repeat with certain periodicities (U). In all other situations, the model is not applicable. Luckily, even within these constraints, the model might nevertheless be helpful in practice, since many drum patterns used as stimuli in music psychological studies correspond to this description or can quite easily be converted to this format, and a majority of listeners has heard this kind of drum patterns in the past.

## 6 Conclusions

This study modelled the complexity of Western popular music drum patterns with three different instrumental voices (bass drum, snare drum, cymbals) based on the ideas of predictive coding, a general theory of perception and learning. To my knowledge, implementing ideas from cognitive theory in this way is a novelty in the modelling of musical complexity. Existing complexity models in the field are usually based on domain-specific music theoretic concepts such as syncopation (see Ref. [9]), or on information theoretic concepts such as Kolmogorov complexity (see Ref. [13]), but not on cognitive theories.

The model involves ideas on enculturation and a Bayesian learning process, which, I think, is a novelty as well. It firstly derives listeners' expectations from a large corpus of drum patterns, thus simulating the enculturation of the listeners (for a similar approach to simulating enculturation in rhythm perception, see Ref. [43]). It secondly uses a short-term learning mechanism that adapts the listeners' expectations at each moment in a pattern based on previous events. Finally, it quantifies surprisal based on the prediction errors and uses surprisal to calculate an estimated complexity score that is compatible with the perceived complexity measures presented in Senn et al. ([22]).

The successful use of predictive coding theory in order to model drum pattern complexity shines a light back on predictive coding. The good fit of the model provides circumstantial evidence to the growing number of studies indicating that predictive coding is not only an elegant and parsimonious theory of the mind and of cognitive processes, but that it also explains empirical findings from behavioural and neurological studies with remarkable success (see Refs. [27,[44], [45], [46]]). In the present study, a rigourous implementation of one basic predictive coding idea led to a very good model relying on the optimisation of only one single parameter. This is a an indirect indication that predictive coding theory in general and the surprisal function in particular indeed grasps essential aspects of perceived drum pattern complexity.

Percepts are often complex in many different ways. Music, for example, may be complex due to its rhythmic properties, but also harmony, melody, polyphony, instrumentation, or any other musical dimension may add to complexity. The complexity of the entire percept can be conceived as an aggregate of all surprises occurring in the different dimensions affecting one single surprisal function in the listener. The aggregation of complexities might even encompass different sensory modalities: both visual and auditory information might affect the perceiver's surprisal function simultaneously and contribute to the complexity of dance or film. Finally, a surprisal-based theory of complexity might also be able to account for *enculturation* (an enculturated perceiver potentially starts from a more accurate internal model, whereas a stranger to the culture might start from an idadequate model) or *domain-specific expertise* (experts might learn faster than non-experts). The surprisal-based theory of perceived complexity may be expanded beyond music to other arts and to further domains of perception, and it might potentially represent the nucleus of a general theory of perceived complexity.

## Author contribution statement

Olivier Senn: Conceived and designed the study; Created the mathematical model; Analyzed and interpreted the data; Contributed materials, analysis tools or data; Wrote the paper.

## Funding statement

This research did not receive any specific grant from funding agencies in the public, commercial, or not-for-profit sectors.

## Data availability statement

Data associated with this study has been deposited at https://zenodo.org/record/7520032#.

## Additional information

No additional information is available for this paper.

## Declaration of competing interest

The author declares that he has no known competing financial interests or personal relationships that could have appeared to influence the work reported in this paper.
